# Medical Induction for Mid trimester Abortion: A Hospital-Based Descriptive Cross-sectional Study

**DOI:** 10.31729/jnma.5502

**Published:** 2020-10-31

**Authors:** Jyotshna Sharma, Sanjeeb Tiwari, Manoj Pokhrel, Lhakpa Lama

**Affiliations:** 1 Department of Obstetrics and Gyanecoiogy, Kathmandu Medical College-Teaching Hospital, Kathmandu, Nepal; 2 Department of General Practice and Emergency Medicine, Maharajgunj Medical Campus, Institute of Medicine, T.U., Maharajgunj, Kathmandu, Nepal

**Keywords:** *abortion*, *dilation and evacuation*, *medical induction*

## Abstract

**Introduction:**

Second trimester abortion is known as termination of pregnancy from 13-28 weeks of gestation which can be further divided into early second trimester as 13-22 weeks and late as 23-28 weeks. In our study we have limited up to early second trimester. We intend to see the success rate of combination of mifepristone and misoprostol for medical induction, median time required for expulsion, complication and need of dilation and evacuation in some cases. This study also aims to give a review of current literature in mid trimester abortion with respect to efficacy, complication and also to provide evidencebase recommendation for safe regimens for mid trimester pregnancy termination.

**Methods:**

This was hospital-based descriptive cross-sectional study conducted among 40 pregnant women at second trimester admitted for termination of pregnancy in Kathmandu medical collage teaching hospital for the period of six month. Ethical approval was taken from the Institutional Review Committee of Kathmandu Medical College (Ref: 2207202002). Convenient sampling was done. All the pregnant women who need to terminate their pregnancy at second trimester (13-22weeks) were admitted at Kathmandu Medical College Teaching hospital for termination of pregnancy were included in the study.

**Results:**

Among the 40 women, who had termination of pregnancy at second trimester 37(92.5%) had successful medical termination whereas 3 (7.5%) needed dilatation and evacuation.

**Conclusions:**

The combination of Mifepristone and Misoprostol have excellent result for termination of pregnancy if appropriately used after evaluating the patient with minimal complications.

## INTRODUCTION

More than one third of pregnancies occurring worldwide are unwanted in which 20% ends up in termination of pregnancy.^1,2^ About 10-15% of abortion happen in second trimester and this accounts for more than two-third of major complication.^3,4,5^ In order to reduce the complications by surgery, the use of mifepristone and misoprostol (MI) has been considered to be successful and effective method for termination of second trimester abortion.^6,7,8^ With the combined regimen, the median time for expulsion is between six and nine hours.^9,10^

Second trimester abortion has been offered in high level facilities by specialists. These factors have resulted in limiting the availability and reach of medical abortion beyond 12 week despite of growing evidence demonstrating to be safe and effective. MI has been considered as effective method for termination of pregnancy which can really help the doctors practicing in remote part of Nepal with minimal resources.

The aim of the study is to study the success rate of combination of mifepristone and misoprostol for medical induction, median time required for expulsion, complication and need of dilation and evacuation in some cases.

## METHODS

This was a hospital-based descriptive cross-sectional study conducted from 1st of October 2019 to 30th of March 2020 for six months period at Kathmandu Medical College Teaching Hospital. Ethical approval was obtained from the Institutional Review Committee of Kathmandu Medical College (Ref: 2207202002). All the pregnant women who need to terminate their pregnancy at second trimester (13-22weeks) were admitted at Kathmandu Medical College Teaching Hospital for termination of pregnancy were included in the study. The convenient sampling method was used. The sample size was calculated by using formula,

n = Z^2^ × p × q / e^2^

Where,

n = Sample size,
Z = 1.96 at 95% Confidence Interval,
p = 0.05,
q = 1-p = 0.85
e = Margin of error, 7%
n = (1.96)^2^ × 0.05 × (1 − 0.05) / (0.07)^2^
  = .182476/.0049 = 37.24

Although sample size calculated was 38, total participants included were 40. Women who came to KMCTH for termination of intrauterine pregnancy of 13-22 weeks by last menstrual period and confirmed by ultrasound were included in the study. Those women with allergies or have contraindication to mifepristone and misoprostol were excluded from the study. Once the women were counselled and consented for termination of pregnancy, informed written consent was taken from the participants. Detailed clinical history was taken and participants were given the one tablet of mifepristone 200 mg to be taken with water and participants were given two tablets of misoprostol 400 mg to be taken after one day sublingually at early morning and asked to come to hospital within 1 hour of taking misoprostol. Once participants arrived at hospital repeat doses of misoprostol was given in three hourly duration from the initial dose of misoprostol till expulsion of fetus and placenta. All women were given medication for pain.

Timing of misoprostol, number of doses of misoprostol required, pain medication, induction to expulsion time and bleeding were noted and filled in the Performa. All data were entered and analyzed using SPSS 17. The descriptive statistical analysis was done; frequency and percentages were calculated for binary variable whereas mean, median and standard deviation were calculated for continuous variable.

## RESULTS

In our study the total of forty women underwent second trimester abortion. The range of age was from 18 to 38 years, mean age being 27.45 years (SD-4.93) (Table 1). The participants were from gravida 1 to 4 and mean being 2.38 (SD-0.868) mode being 2. Ninety five percent (n = 38) of women are multipara. Among 40 women 4 (10%) had previous experience of abortion, 6 (15%) had previous one cesarean section for prior delivery. The gestational age of the participants was from 13-20 weeks and mean being 15.435 (SD-1.889). Minimal dose of Misoprostol was 1 and 5 being the maximal dose, mean being 2.48 (SD-0.751). After 2 doses of Misoprostol 22 (55.0%) of participant had medical induction.

**Table 1:** Descriptive Statistics on abortion, gestational age and misoprostol dose.

S.N		Age	Gravida	Parity	Abortion	Gestational Age	Misoprostol Dose	Time Duration
1.	N Valid	40	40	40	40	40	40	37
2.	Missing	0	0	0	0	0	0	3
3.	Mean	27.45	2.38	.10	1.80	15.453	2.48	7.215
4.	Median	26.00	2.37	.10	1.67	15.375	2.44	7.417
5.	Mode	23	2	0	1	13	2	8
6.	S. D	4.930	.868	.304	.939	1.8892	.751	2.8015
7.	Minimum	18	1	0	1	13	1.751	2
8.	Maximum	38	4	1	4	20	5	15

The major cause of termination was mental health which was 20 (50.0%), followed by fetal anomaly 10 (25.0%), medical condition being 8 (20.0%) and rape/ incest was 2 (5.0%) (Table 2).

**Table 2:** Causes of Termination of Pregnancy.

S.N	Causes	n (%)
1.	Mental Health	20 (50)
2.	Fetal anomaly	10 (25)
3.	Medical condition	8 (20)
4.	Rape/ incest	2 (5)
	Total	40 (100)

Most of the participant had medical induction 37 (92.5%) and 3 (7.5%) medical induction were converted to D & E after second dose of Misoprostol due to heavy bleeding. Time duration for medical induction was from 2 hours to 15 hours. Mean time duration was 7.215 hours (SD-2.8015) (Table 3).

**Table 3:** Time Duration From Medical Induction to Abortion.

S.N	Time Duration (Hours)	n (%)
1.	2.0	1(2.5)
2.	2.5	1 (2.5)
3.	4.0	3 (7.5)
4.	4.5	3 (7.5)
5.	5.0	4 (10.0)
6.	5.5	1 (2.5)
7.	6.5	1 (2.5)
8.	7.0	4 (10.0)
9.	7.5	2 (5)
10.	8.0	7 (17.5)
11.	8.5	2 (5.0)
12.	9.5	2 (5.0)
13.	10.0	2 (5.0)
14.	11.0	2 (5.0)
15.	13.0	1 (2.5)
16.	15.0	1 (2.5)
17.	Total	37 (92.5)
18.	Medical Induction Converted to Dilatation and Evacuation (D and E)	3 (7.5)
19.	Grand Total	40 (100.0)

## DISCUSSION

Mifepristone and Misoprostol has been used for medical induction in different regime and interval. In our study following the latest WHO recommendation Mifepristone 200 milligram was given to all the participants and Misoprostol 400 microgram was given at three hours interval till expulsionfor second trimester medical abortion.

Aniteye etal, had stated two third of major complication occurs during medical abortion in second trimester abortion but in our study, it was 3 cases (7.5%) who had bleeding. Mentula, et al. had found medical abortion to be safe and D & E in around one third of the cases in second trimester abortion as compared to our study the abortion was safe and D & E was in7.5% of the cases. Gemzell, et al. had recommended combination of mifepristone and misoprostol and its effectiveness in second trimester abortion in their study, like wise in our study we use combination of mifepristone and misoprostol and it was 92.5% effective in medical induction. Ulmann, et al. had success rate of 95.3% of medical induction as compared to our study,which was 92.5%. Dabash, R., et al. recorded 91.7% complete uterine evacuation after using combination of mifepristone and misoprostol as compared to our study where it was 92.5%.

Karki, et al. in their study the age of women undergoing second trimester abortion was 26-30 years, 81.11% of the women had never undergone abortion, mental cause appeared to be the major reason for abortion constituting 82.04%, success was 90.58%, expulsion with total five doses of misoprostol and induction to abortion time was 4-7 hours11 which was comparable to our study, mean age was 27.45 years, 90% had never undergone abortion, 50% was mental cause, success rate was 92.5% and expulsion with total of five doses of misoprostol and induction to mean abortion time was 7.215 hours.

The limitations of the study are: being single centre study, the results can not be generalized; being cross-sectional study, the association to the cause factor can not be determined and biases can be possible.

## CONCLUSIONS

The combination of Mifepristone and Misoprostol have excellent result for termination of pregnancy if appropriately used after evaluating the patient with minimal complications.

## Conflict of Interest

**None.**
